# A rapid high-resolution method for resolving DNA topoisomers

**DOI:** 10.1186/s13104-018-3147-6

**Published:** 2018-01-16

**Authors:** Lesley A. Mitchenall, Rachel E. Hipkin, Michael M. Piperakis, Nicolas P. Burton, Anthony Maxwell

**Affiliations:** 1 Department of Biological Chemistry, John Innes Centre, Norwich Research Park, Norwich, NR4 7UH UK; 2 Qiagen Ltd., Skelton House, Lloyd St. North, Manchester, M15 6SH UK; 3 Present Address: Fluidigm Ltd, 12 New Fetter Lane, London, EC4A 1JP UK; 4 Present Address: University Centre, Blackburn College, University Close, Blackburn, Lancashire BB2 1LH UK; 5 Inspiralis Ltd, Innovation Centre, Norwich Research Park, Colney Lane, Norwich, NR4 7UH UK

**Keywords:** Capillary electrophoresis, DNA topoisomerases, Plasmids, Minicircles

## Abstract

**Objective:**

Agarose gel electrophoresis has been the mainstay technique for the analysis of DNA samples of moderate size. In addition to separating linear DNA molecules, it can also resolve different topological forms of plasmid DNAs, an application useful for the analysis of the reactions of DNA topoisomerases. However, gel electrophoresis is an intrinsically low-throughput technique and suffers from other potential disadvantages. We describe the application of the QIAxcel Advanced System, a high-throughput capillary electrophoresis system, to separate DNA topoisomers, and compare this technique with gel electrophoresis.

**Results:**

We prepared a range of topoisomers of plasmids pBR322 and pUC19, and a 339 bp DNA minicircle, and compared their separation by gel electrophoresis and the QIAxcel System. We found superior resolution with the QIAxcel System, and that quantitative analysis of topoisomer distributions was straightforward. We show that the QIAxcel system has advantages in terms of speed, resolution and cost, and can be applied to DNA circles of various sizes. It can readily be adapted for use in compound screening against topoisomerase targets.

## Introduction

Agarose gel electrophoresis has been the method of choice for separating DNA molecules of moderate size (~500 bp–50 kbp) since the 1970s [1]. The mobility of linear DNA molecules is generally dependent on their size and can be used to estimate the sizes of the products of restriction enzyme digestions. Further, the method can be extended to the separation of different forms of circular DNA (e.g. supercoiled, relaxed and nicked) [2, 3]; the relative mobility varying with the gel percentage and voltage gradient [4]. Although the resolution of DNA topoisomers is limited in standard gels this can be enhanced by the inclusion of intercalating agents (ethidium bromide (EtBr) and chloroquine), which alter the twist of the DNA and lead, in closed-circular double-stranded DNA molecules, to changes in writhe that will enable topoisomers unresolvable under native conditions to be visualised [5]. Further enhancement of the resolving power of agarose gels can be achieved by two-dimensional electrophoresis in which, typically, topoisomers are first run in one dimension with no, or low, intercalator, and then rotated through 90° and run in the second dimension in the presence of a higher concentration of the intercalator [6].

An important application of agarose gel electrophoresis is in the analysis of the reactions of DNA topoisomerases, enzymes that catalyse topological changes in DNA [7, 8]. Typically, these enzymes are assayed by monitoring the interconversion of relaxed and supercoiled plasmid DNAs. Topoisomerases are regarded as important targets for both antibacterial and anti-cancer agents and the assays can be used to assess the inhibition of their activity by these compounds [9, 10].

Despite the utility of gel electrophoresis in studying the activities of topoisomerases and other enzymes, the technique suffers from several drawbacks. Firstly, gel electrophoresis is relatively slow, typically taking an hour or more to complete. Secondly, the process is intrinsically low-throughput, generally involving manual-handling steps, although a degree of automation in terms of liquid-handling devices, etc. is possible. Thirdly, agarose gel electrophoresis is relatively costly, especially if carried out on a large scale; and finally, there are potential safety issues with the use of chloroquine and EtBr. In part to circumvent these issues, alternative methods have been developed.

Different topological forms of DNA can be separated by CsCl isopycnic centrifugation, and visualised by microscopy methods (electron microscopy, cryo-EM and atomic force microscopy) but these methods are unsuitable for routine assays. Alternative analytical methods based on DNA triplex formation have been developed. These exploit the observations that, under certain conditions, different topological species form intermolecular triplexes more efficiently. In one application of this method, a triplex-forming oligonucleotide is tethered to microtitre plate wells and more negative supercoiled DNA species are selectively captured and subsequently detected using a fluorescent dye [11]; this assay can be used in a high-throughput mode to discover topoisomerase inhibitors [12]. In an alternative version, an oligonucleotide is labelled with a fluorescent dye and the readout is fluorescence anisotropy [13]; in this case, triplex formation occurs preferentially with relaxed DNA. A novel method for resolving topoisomers of DNA has been described involving quinine carbamate ligands attached to a silica gel [14]. This matrix can be utilised to achieve rapid and efficient preparation of DNA topoisomers. However, it is not clear whether this method can be utilised in routine assays.

Capillary electrophoresis is a sensitive and versatile technique suitable for the analysis of a wide range of analytes including large biomolecules, such as proteins and nucleic acids [15]. It works by separating ions based on their electrophoretic mobility with the use of an applied voltage and is often performed in sub-millimeter diameter capillaries and micro- and nanofluidic channels. The separation of covalently-closed and open-circular DNA forms with capillary electrophoresis has been described previously [16], but only plasmid pUC18 was examined and the resolution of only a few topoisomers was reported. In addition, the use of capillary gel electrophoresis with laser-induced fluorescence has been described for the detection of linear, open circle, and supercoiled plasmid DNAs [17]. In this case, the resolution of relaxed DNA (from the open circle form) and the separation of individual topoisomers of different linking numbers were not reported. In neither case was the technique suitable for use in a high-throughput format [16, 17]. Despite these previous reports, the use of capillary gel electrophoresis has not been widely adopted for analysing topoisomers of circular DNAs and gel electrophoresis remains the predominant analytical method.

We have assessed a number of commercial capillary electrophoresis systems for their ability to resolve DNA topoisomers and have found that the QIAxcel Advanced System is well-suited to this purpose. This system has been developed as a benchtop multichannel capillary electrophoresis device for the analysis of DNA and RNA samples [18]. The QIAxcel Advanced System performs sensitive, high-resolution capillary electrophoresis of up to 96 samples per run. In comparison with agarose gel electrophoresis, samples can be smaller in volume, take less time to run and have better resolution without the addition of extra, possibly mutagenic, chemicals; the gel matrix is contained and exposure to hazardous material is minimal. This instrument completely automates nucleic acid separation, analysing 12 samples at a time in a few minutes. It is designed for medium-to high-throughput analysis of linear DNA PCR products or restriction enzyme digests, but here we show that it can be used to resolve double-stranded circular DNA molecules of the same molecular weight that differ only in topology.

## Main text

### Methods

#### Materials

Supercoiled plasmids pBR322 and pUC19 were supplied by Inspiralis Ltd (Norwich, UK). A 339 bp minicircle was prepared as described previously [19]. DNA gyrase was made and assayed as described previously [20, 21]. Ciprofloxacin was purchased from Sigma.

#### Preparation of topoisomers

**Fig. 1:**
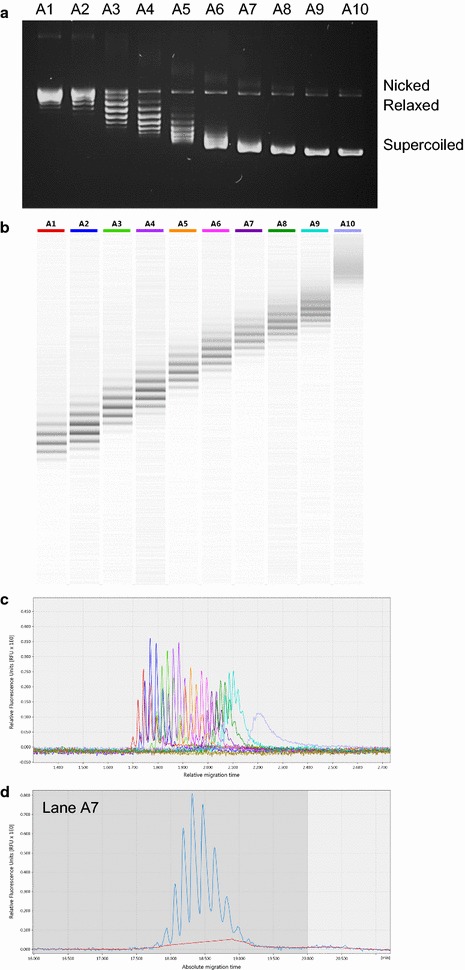
Separation of topoisomers of plasmid pBR322 by agarose gel electrophoresis and QIAxcel Advanced System. **a** 1% agarose gel, stained with EtBr, of plasmid pBR322 relaxed by human topo I in the presence of the following concentrations of EtBr (µg/mL): A1–0; A2–0.002; A3–0.004; A4–0.006; A5–0.008; A6–0.010; A7–0.012; A8–0.014; A9–0.016; A10–0.02. The same samples were resolved by the QIAxcel system and are depicted as a digital gel image (**b**) and an electropherogram (**c**); colours in (**b**) match those in (**c**). **d** Individual densitometric trace of sample A7 from (**c**) illustrating the resolution of the topoisomers

Supercoiled plasmid pBR322 was incubated with human DNA topoisomerase (topo) I (Inspiralis Ltd) and various concentration of EtBr (Sigma; 0–0.2 µg/mL) (Fig. 1), following published methods [22, 23]. Reactions containing 20 mM Tris·HCl (pH 7.5), 200 mM NaCl, 0.25 mM EDTA, 5% glycerol with 2.5 µg of DNA in a 50 µL volume reaction, were incubated for 90 min at 37 °C. Reactions were diluted to 100 µL with 10 mM Tris·HCl (pH 7.5), 1 mM EDTA, then extracted twice with phenol/chloroform/isoamyl alcohol (25:24:1) followed by a chloroform/isoamyl alcohol (24:1) extraction, ethanol precipitation and washing with ice-cold 70% ethanol; pellets were resuspended in 15 µL 10 mM Tris·HCl (pH 7.5), 1 mM EDTA. Topoisomers of pUC19 were prepared by the same method. Topoisomers of the 339 bp minicircle were prepared using a similar method, as described previously [19].

#### DNA gyrase supercoiling reactions

Relaxed plasmid pBB322 (Inspiralis Ltd) was incubated with *Escherichia coli* DNA gyrase as described previously [21]. The reaction was inhibited by the addition of various amounts of ciprofloxacin (see Fig. 3) and the products analysed by agarose gel electrophoresis and capillary electrophoresis.

#### Gel electrophoresis

**Fig. 2:**
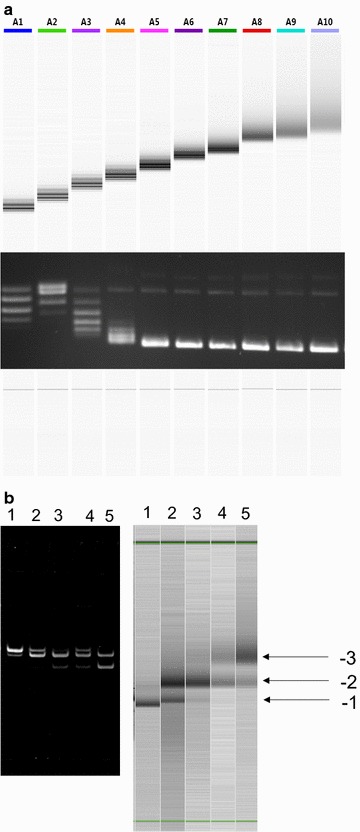
Separation of topoisomers of plasmid pUC19 and a 339 bp circle. **a** Samples of pUC19 of varying linking number, prepared as in Fig. 1. The upper panel is a digital gel image; lower panel shows a 1% agarose gel. **b** Samples of a 339 bp minicircle of varying linking number; left panel shows a 5% polyacrylamide gel (in 40 mM Tris·acetate [pH 8.0], 10 mM CaCl_2_); the right panel is a digital gel image

**Fig. 3:**
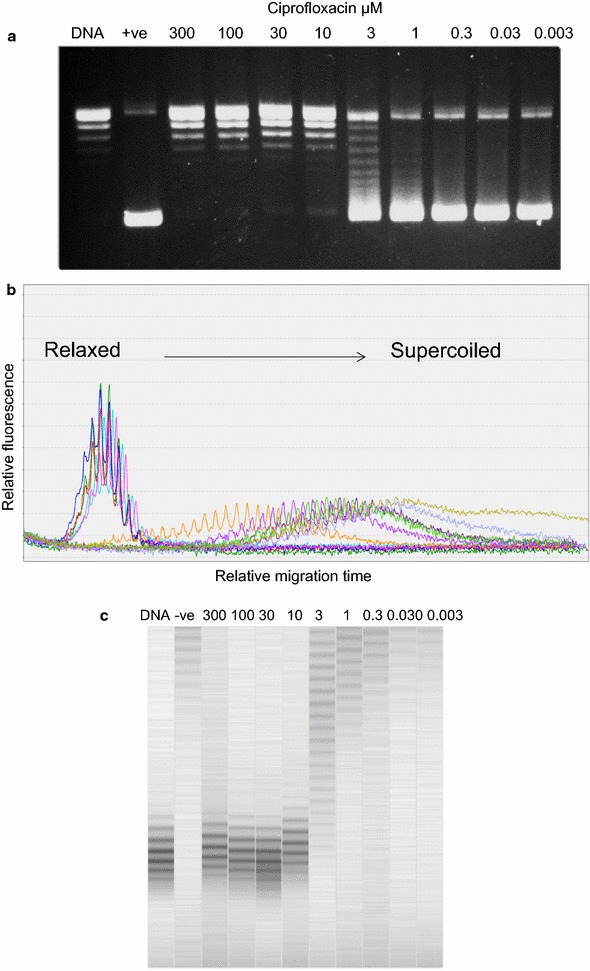
Inhibition of DNA gyrase-catalysed supercoiling by ciprofloxacin. **a** 1% agarose gel, stained with EtBr, showing plasmid pBR322 being supercoiled by DNA gyrase, and inhibition of this reaction by ciprofloxacin. DNA indicates relaxed DNA alone; +ve indicates gyrase only; all other tracks contain gyrase and the amounts of ciprofloxacin (in µM) indicated. **b** Electropherogram of the same samples as in (**a**); colours indicate the individual samples from (**a**). **c** Digital gel image of the same samples

To separate plasmid topoisomers, samples (15 µL) containing 150 ng (10 ng/µL) DNA were mixed with an equal volume of 40% sucrose, 100 mM Tris·HCl (pH 8.0), 100 mM EDTA, 0.5 mg/mL bromophenol blue and run on a 1% agarose (Melford) gel in 40 mM Tris, 20 mM acetic acid, 1 mM EDTA, pH 8.0 at 80 V for 2 h. To separate topoisomers of the 339 bp minicircle, samples were applied to a 5% polyacrylamide gel (in 40 mM Tris·acetate [pH 8.0], 10 mM CaCl_2_), as described previously [19]. (The results reported in Figs. 1, 2, 3 were repeated independently at least three times.)

#### Capillary electrophoresis

DNA samples (10 µL of 10 ng/µL DNA) without loading buffer were placed into the QIAxcel Advanced instrument and separated using the QIAxcel DNA High Resolution Cartridge with the pre-set OM1200 method, which includes the following electrophoresis parameters: alignment marker injection at 4 kV for 10 s, sample injection at 5 kV for 5 s and separation at 3.5 kV for 1200 s; the QX Alignment Marker 15 bp/10 kb was run simultaneously with the samples. Separation took ~ 25 min. (Capillary electrophoresis separations reported in Figs. 1, 2, 3 were replicated at least three times.)

## Results and discussion

To assess the ability of the QIAxcel system to resolve different topological forms of closed-circular DNA, we generated a set of plasmid pBR322 samples (4361 bp) with a wide distribution of linking numbers. Figure 1 shows a comparison of these samples analysed on a 1% agarose gel (Fig. 1a) and using the QIAxcel System (Fig. 1b–d). The output from the QIAxcel Screengel^®^ software can be displayed as a digital gel image (Fig. 1b) and an electropherogram (Fig. 1c). We found that the QIAxcel system is capable of very good resolution of DNA topoisomers (Fig. 1d) that differ in linking number by one over a wide range. As can be seen from Fig. 1, agarose gel electrophoresis is able to resolve ~ 10 topoisomers under these conditions. In contrast, the QIAxcel system can resolve at least 22 topoisomers. Further resolution using agarose gel electrophoresis could only be achieved by running further gels in the presence of chloroquine or by using two-dimensional electrophoresis. In Fig. 1b, c only sample A10 (the most highly negatively supercoiled) was poorly resolved under these conditions; better resolution of more highly supercoiled topoisomers can be achieved by adjusting the running conditions. The electropherogram can be interrogated in several ways and the software allows manual integration of digitally displayed Gaussian distributions of topoisomers (Fig. 1d). Such data can be re-plotted to determine K (the elastic constant), Lk^0^ (mean linking number) and ω (the angular displacement between the most intense topoisomer, Lk_m_, and Lk^0^), as described elsewhere [6] (data not shown).

We have also utilised the QIAxcel system with a smaller plasmid (pUC19; 2686 bp) and a small (339 bp [19]) DNA circle and achieved similar results (Fig. 2). In the case of pUC19, ~ 6 topoisomers can be resolved by agarose gel electrophoresis, compared with ~ 16 using the QIAxcel system. For the 339 bp circle only three topoisomers are seen (due to its small size); these cannot readily be resolved using agarose gel electrophoresis; in this case polyacrylamide gel electrophoresis was used, which achieved similar resolution to the QIAxcel system.

In other experiments we utilised the QIAxcel system to monitor supercoiling by DNA gyrase and the inhibition of this reaction by the quinolone drug ciprofloxacin (Fig. 3). We found that the QIAxcel system can resolve topoisomers formed during a gyrase supercoiling reaction and can readily be used to determine the IC_50_ value (the concentration of the inhibitor where supercoiling activity is reduced by half), which was 3 µM in this case. In these experiments we have shown that the QIAxcel cartridge can withstand the inclusion of up to 10% DMSO with little detriment to overall resolution.

## Conclusion

We have shown that the QIAxcel system can be used to resolve DNA topoisomers of circles of a range of sizes: 339–4361 bp. The range of topoisomers that can be resolved in a single run for plasmids pBR322 and pUC19 greatly exceeds that achievable using agarose gel electrophoresis. Moreover, the speed of the process exceeds that achievable using agarose gels. The digitisation of the data means that the results can be outputted in a variety of formats and can readily be quantitatively analysed. These results suggest that the QIAxcel system can be used for the analysis of the topology of closed-circular DNAs and for analysing the kinetics of topoisomerase reactions, and could readily be adapted to be utilised in medium- to high-throughput analysis of DNA samples in topoisomerase drug-discovery experiments.

## Limitations

There are both advantages and potential disadvantages in using the QIAxcel system. The QIAxcel system has advantages in terms of speed, the requirement for limited amounts of DNA samples, and in terms of the data output and ease of analysis. In principle it is more cost-effective than agarose gel electrophoresis in terms of consumables but, it does require capital outlay in terms of the necessary instrumentation. In addition, we have found that, for this application, optimisation of the running conditions for different types of samples is necessary, which could be a time-consuming process, although this can also be the case with conventional gel electrophoresis.

## Abbreviations

DMSO: dimethyl sufloxide
EtBr: ethidium bromide
